# Prognostic significance of factor XIIIA promoter methylation status in aneurysmal subarachnoid haemorrhage (aSAH)

**DOI:** 10.1186/s12872-019-1146-8

**Published:** 2019-07-17

**Authors:** S. Arati, G. K. Chetan, M. K. Sibin, Dhananjaya I. Bhat, Vikas Vazhayil, K. V. L. Narasingarao

**Affiliations:** 1 Department of Human Genetics, National Institute of Mental Health and Neuro Sciences, Bangalore/Karnataka, Karnataka 560029 India; 2 Department of Biochemistry, Armed Forces Medical College, Pune, 411040 India; 3 Department of Neurosurgery, National Institute of Mental Health and Neuro Sciences, Bangalore, 560029 India

**Keywords:** Aneurysmal subarachnoid haemorrhage, Apolipoprotein E, Factor XIII, Promoter methylation

## Abstract

**Background:**

Aneurysmal subarachnoid hemorrhage is a life- threatening condition with high rate of disability and mortality. Apolipoprotein E (*APOE*) and Factor XIIIA (*F13A*) genes are involved in the pathogenetic mechanism of aneurysmal subarachnoid haemorrhage (aSAH).

We evaluated the association of promoter methylation status of *APOE* and *F13A* gene and risk of aSAH.

**Methods:**

For evaluating the effect of hypermethylation in the promoter region of these genes with risk of aSAH, we conducted a case -control study with 50 aSAH patients and 50 healthy control. The methylation pattern was analysed using methylation specific PCR. The risk factors associated with poor outcome after aSAH was also analysed in this study. The outcome was assessed using Glasgow outcome score (GOS) after 3 months from the initial bleed.

**Results:**

The frequency of *APOE* and *F13A* methylation pattern showed insignificant association with risk of aSAH in this study. Gender stratification analysis suggests that *F13A* promoter methylation status was significantly associated with the risk of aSAH in male gender. Age, aneurysm located at the anterior communicating artery and diabetes mellitus showed significant association with poor outcome after aSAH.

**Conclusion:**

There was no significant association with *APOE* promoter methylation with the risk as well as outcome of patients after aSAH. *F13A* promoter methylation status was significantly associated with risk of aSAH in male gender, with no significant association with outcome after aSAH.

## Background

Aneurysmal subarachnoid hemorrhage (aSAH) is a worldwide disease condition which causes permanent disability and high rate of mortality [1]. The worldwide incidence rate of SAH is about 9/ 100,000 persons/year with regional differences. Most important risk factors for the rupture of aneurysm were female gender, hypertension, size and location of aneurysm [2]. Epigenetic mechanisms such as DNA methylation, histone modification and RNA Interference were associated with aging mechanisms and cause risk for cardiovascular and hemorrhagic stroke [3]. In DNA methylation, the cytosine at the CpG islands in the promoter region is methylated at the 5′ carbon position by DNA methyltransferase (DNMT). DNA methylation is associated with long term gene silencing without altering nucleotide sequence of the DNA [4]. The prognosis of SAH depends on the amount of initial bleed, re-bleeding, chance of delayed cerebral ischemia, stress hyperglycemia, and elevated d-dimer levels [5].

In CNS, Apolipoprotein E *(APOE*) protein is synthesized by astrocytes. The main function is to transport cholesterol and triglycerides. *APOE* is involved in pathogenic mechanism of aneurysm formation by regulating inflammatory responses [6] and triggering atherosclerosis [7]. *APOE* gene is in the chromosome 19q13.The gene consists of four exons and three introns which gives mRNA of 1163 nucleotides [8]. The promoter region of *APOE* gene spanning from − 1017 to + 406 has three major SNP [9] and contributed to a major risk factor for Alzheimer’s disease in Italian case -control study [10]. There was a functional difference between *APOE* 3′ and 5′ CpG islands. The 3’CpG islands were methylated in all tissues expect sperm and was not repressed whereas the 5’CpG islands methylation led to transcriptional repression [11]. The GC content of *APOE* promoter region was about 58%, a minimum content of 42% and a maximum of 82% [12].

Factor XIIIA (*F13A*), also called as fibrin stabilizing factor, is an important enzyme in the blood coagulation system [13]. The main function of *F13A* is to cross link fibrin to form the final blood clot [14]. The *F13A* gene is located in the chromosome 6p25.1 [15]. The hyper methylation of *F13A* promoter region repress the gene transcription and the deficiency of *F13A* causes life threatening central nervous system bleeding [16]. Fibrinolytic system dysfunction is another pathogenic mechanism that contributes to aSAH [17, 18]. *F13A* is an important enzyme in the fibrinolytic system. It was reported that fibrinolytic system interacts with components of extra cellular matrix remodelling and contributes to the risk of haemorrhage [19, 20].

There are many studies which describes the association of *APOE* and *F13A* polymorphism and the risk of aSAH. But studies which explains the role of epigenetic mechanisms in *APOE* and *F13A* genes with the risk of aSAH are very less. In the present study, we have analysed the association of promoter methylation status of *APOE* and *F13A* gene with the risk of aSAH and its association with the outcome of patients after aSAH.

## Methods

### Study population

The blood samples of 50 aSAH patients and 50 age and sex matching healthy controls were included in the study. The characteristics of study population were already published previously (DOI: 10.1186/s12881-018-0674-x). The WFNS scale is widely used to classify patients based on Glasgow coma scale (GCS). WFNS grade II has GCS of 14 in the absence of motor deficit whereas WFNS grade III has GCS of 13 with the presence of motor deficit. Outcome was assessed using Glasgow outcome score (GOS) after 3 months from the initial bleed. Poor outcome was defined according to GOS as severe or worse disability.

### DNA isolation, bisulfite modification, methylation specific PCR (MS-PCR)

Two millilitre blood was taken from all the participants and DNA was isolated from each blood sample using the commercially available Machery-Nagel (MN) kit according to manufacturer’s protocol. Purity and quantity of DNA was analysed by Nanodrop ND2000c spectrophotometer. DNA with a purity of 1.75–1.85 was used for Bisulfite modification. 2 μg of DNA was isolated from blood and was bisulfite modified using EpiTech Bisulfite Kit (Qiagen, Valencia, CA, USA) according to the manufacturer’s protocol. 200 bp and 100 bp fragment of *APOE* and *F13A* promoter was amplified by MS PCR from bisulfite treated DNA using methylated and unmethylated primers. Methyl primer express software, version 1.0 (Applied Biosystems) was used to design the methylated and unmethylated primers for *APOE* and *F13A* promoters as shown in Table 1. The reaction for MS -PCR was prepared for a final volume of 10 μl, which contain 5 μl PCR master mix (EmeraldAmp GT 2X), 2 μl primer (2 μm),2 μl DNA (2 μg),1 μl distilled water. The MS -PCR conditions were initial incubation at 95^0^ C for 10 min followed by 40 cycles of denaturation at 95 °C for 30 s, annealing at 56 °C (*APOE* methylated and unmethylated primers) for 22 s, annealing at 58 °C (*F 13A* methylated and unmethylated primers) for 32 s and extension at 72 °C for 30 s. The PCR products were electrophoresed on 2% agarose gel which was stained with ethidium bromide and was visualized under gel documentation system (Biorad, GelDoc EZ imager).

**Table 1:** Primer Sequences and Annealing Temperatures

Gene	Sequence (5′-3′)	Annealing temperature	Product size
*APOE* M	5′ GTTGGGGTTAGTTGATGTTTATTAC 3′ (FORWARD) 5′ AAAAAAACTAAACTCCTAATTCGAA 3′ (REVERSE)	56 °C	200 bp
*APOE* UM	5′ GGTTGGGGTTAGTTGATGTTTATTAT 3′ (FORWARD) 5′ AAAAAAACTAAACTCCTAATTCAAA 3′ (REVERSE)	56 °C	200 bp
*FXIII* M	5′ AGAAGCGAGGGTTTTTGTTC 3′ (FORWARD) 5′ AACAATATAACGCGCTATCG 3′ (REVERSE)	58 °C	100 bp
*FXIII* UM	5′ TAGAAGTGAGGGTTTTTGTTT 3′ (FORWARD) 5′ AACAATATAACACACTATCAAAA 3′ (REVERSE)	58 °C	100 bp

### Statistical analysis

The continuous variable was expressed as mean ± SD. The normal distribution of data was checked using Shapiro-Wilk test. Categorical data were tested using Pearson’s χ^2^. The odds ratio with 95% confidence interval (CI) was estimated to assess the methylation status and risk of aSAH. The interaction of various risk factors and poor outcome at 3 months after aSAH was analysed with logistic regression analysis for estimating the univariate and multivariate odds ratio and 95% CI. All statistical analysis was done using R.3.0.11 statistical software. The *P* value < 0.05 was considered as statistically significant.

## Results

**Fig. 1:**
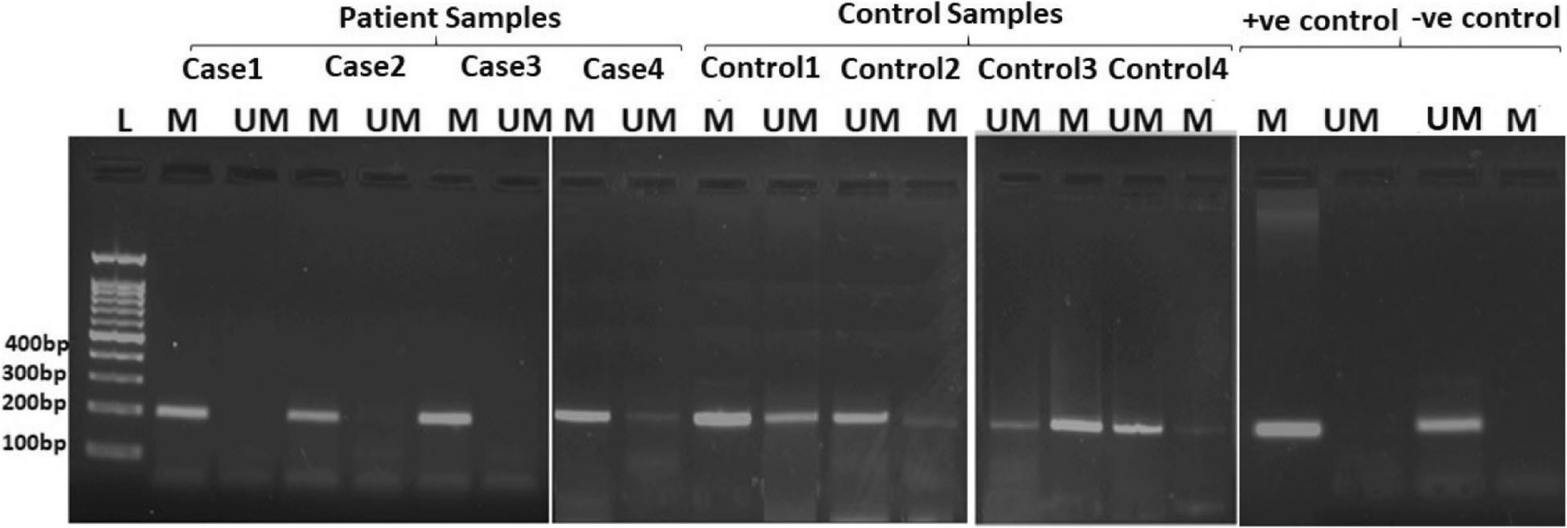
Analysis of methylation status of *APOE* promoter region by MS-PCR of aSAH patients and control. L: Ladder. M: Methylated promoter region of *APOE* gene. UM: Unmethylated promoter region of *APOE* gene. +ve Control: Universally methylated DNA. -ve Control: Universally unmethylated DNA

There was no significant difference in age, gender, hypertension and diabetes mellitus between aSAH patients and control. The demographic characteristics of patients and controls are shown in Table 2. The methylation frequency for *APOE* gene was 64% for aSAH patients and 60% for controls. There was no statistically significant difference in methylation and unmethylation pattern in the promoter region of *APOE* gene between cases and control (*p* = 0.836). The promoter methylation analysis of *APOE* gene in aSAH patients and controls are shown in Table 3. Figure 1 represents the methylation status of *APOE* promoter region by MS -PCR of aSAH patients and healthy controls.

**Table 2:** Demographic characteristics of aSAH patients and healthy controls

Variables	Patients(50)	Control(50)	*P* Value
Age	51.58 ± 10.41	56.64 ± 13.67	0.07
Gender (Male/Female)	19/31	20/30	0.83
Hypertension (Yes/No)	28/22	34/16	0.21
Diabetes mellitus (Yes/No)	27/23	21/29	0.23
Alcohol consumption (Yes/No)	15/35	20/30	0.29
Smoking (Yes/No)	11/39	18/32	0.12
Site of aneurysm (%)			
ACOM*	23	/	
PCOM*	4	/	
ICA*	9	/	
MCA*	5	/	
Multiple	9	/	
Size of aneurysm (%)			
Small(< 15 mm)	38	/	
Large(15-25 mm)	10	/	
Giant(> 25 mm)	2	/	
WFNS Grade (%)			
Grade II	25	/	
Grade III	25	/	

^*^ACOM anterior communicating artery, *PCOM* posterior communicating artery, *MCA* middle cerebral artery, *ICA* internal carotid artery

**Table 3:** Promoter Methylation Analysis of *APOE* and Factor XIII gene in aSAH patients and healthy controls

Characteristics	Methylated (%)	Unmethylated (%)	*p- value*
*APOE*			
All			
Cases(n = 50)	32 (64)	18 (36)	0.836
Control (n = 50)	30 (60)	20 (40)	
Females			
Cases(*n* = 31)	19	12	0.90
Control(*n* = 30)	18	12	
Males			
Cases (*n* = 19)	13	6	0.830
Controls (*n* = 20)	12	8	
*F13A*			
All			
Cases (n = 50)	37 (74)	13 (26)	0.283
Control (*n* = 50)	31 (62)	19 (38)	
Females			
Cases(n = 31)	23	8	0.90
Control(n = 30)	23	7	
Males			
Cases (n = 19)	14	5	0.07
Controls (n = 20)	8	12

**Fig. 2:**
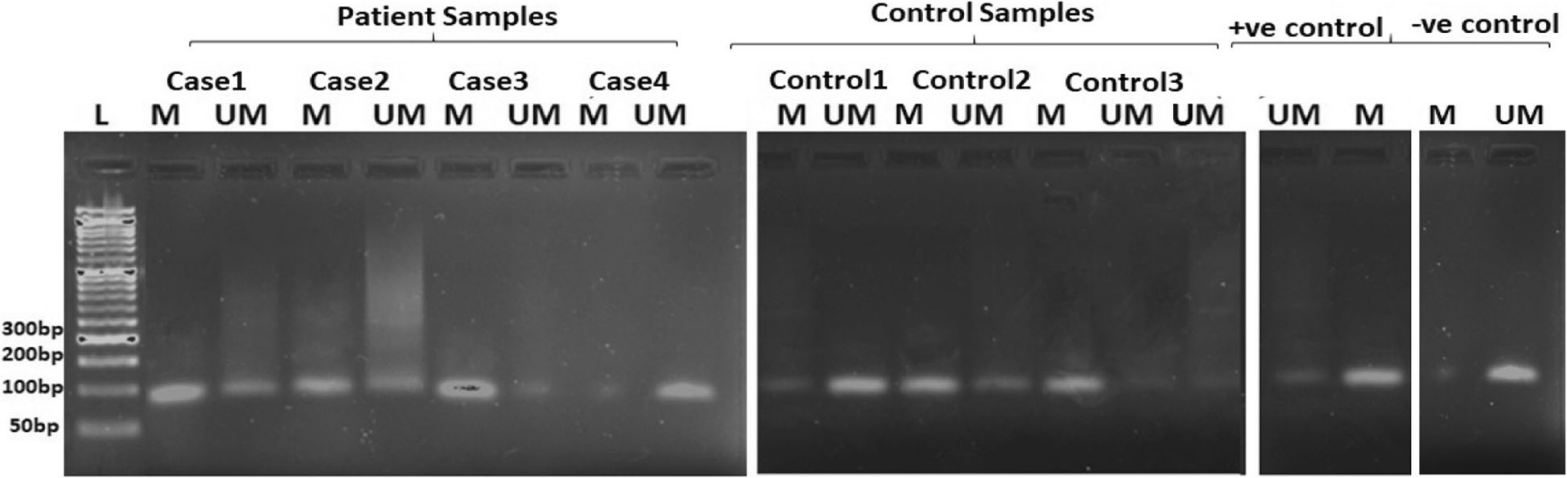
Analysis of methylation status of *F13A* promoter region by MS-PCR of aSAH patients and control. L: Ladder. M: Methylated promoter region of *F13A* gene. UM: Unmethylated promoter region of *F13A* gene. +ve Control: Universally methylated DNA. -ve Control: Universally unmethylated DNA

Likewise, the methylation frequency of *F13A* gene was 74% for aSAH patients and 62% for controls. The promoter methylation and unmethylation pattern of *F13A* gene between cases and control had no statistically significant difference (*p* = 0.283). The promoter methylation analysis of *F13A* gene in aSAH patients and controls are shown in Table 3. Figure 2 represents the analysis of methylation status of *F13A* promoter region by MS -PCR of aSAH patients and healthy controls.

The promoter methylation and unmethylation status of *APOE* (OR = 1.35;95%CI = .58–3.16; *p* = 0.483) and *F13A* (OR = 1.37;95%CI = 0.53–.3.53;*p* = 0.511) genes were not significantly associated with the risk of aSAH after adjustment for covariates as shown in Table 4. Gender stratification analysis showed that *F13A* promoter methylation status was significantly associated with risk of aSAH in male gender (OR = 4.2;95% CI = 1.08–16.32;*p* = 0.03).

**Table 4:** Risk of aSAH based on gene promoter methylation status

Genes	Methylation Status	Crude OR (95% CI)	*p-*value	Adjusted OR^a^ (95%CI)	*p-*value
*APOE*	U (reference)	1.19 (0.52–2.66)	0.680	1.35 (0.58–3.16)	0.483
	M				
*F13A*	U (reference)	1.74 (0.74–4.09)	0.200	1.37 (0.53–3.53)	0.511
	M				

*OR* odds ratio, *U* unmethylation, *M* methylation

^a^Adjusted for smoking, alcohol consumption, hypertension and diabetes

Univariate and multivariate risk factors for poor outcomes are shown in Table 5. Both univariate and multivariate analysis showed that patients with aneurysm located at anterior communicating artery (ACOM) (OR = 0.15,95%CI =0.04–0.52,*P* = 0.0028) and diabetes mellites (OR = 3.75, 95%CI = 1.16–12.1,*P* = 0.03) have association with poor outcome independent of other factors. Also, our study showed age ≥ 50 years can be one of the risk factors for poor outcome in both multivariate and univariate analysis. Of the subjects with *APOE* promoter methylation status (*n* = 32), 50% had good outcome and 50% had poor outcome. Likewise, the *F13A* promoter methylation subjects (*n* = 37), 54% had good outcome and 45% had poor outcome. The promoter methylation status of *APOE* and *F13A* were not associated with outcome either in univariate or multivariate analysis.

**Table 5:** Risk factors for poor outcome after aSAH

Characteristics	Univariate OR(95%CI)	*p*	Multivariate OR(95%CI)	*p*
Age (≥50& ≤ 50)	6.08 (1.71–21.49)	0.0051	5.29 (1.02–27.50)	0.047
WFNS grade (2&3)	1.00 (0.33–3.03)	1.00	–	–
Location				
ACOM*	0.15 (0.04–0.52)	0.0028	0.06 (0.009–0.38)	0.003
PCOM*	3.00 (0.29–31.00)	0.35	–	
MCA*	4.18 (0.43–40.40)	0.216	–	
ICA*	2.58 (0.58–11.40)	0.21	–	
MULTIPLE	2.10 (0.46–9.56)	0.337	–	
Size				
Small	1.73 (0.46–6.43)	0.41	2.30 (0.35–15.00)	0.38
Large	0.54 (0.13–2.23)	0.40	–	–
Giant	0.92 (0.05–15.60)	0.95	–	–
Sex	1.04 (0.33–3.27)	0.94	–	–
Hypertension	0.60 (0.19–1.85)	0.38	–	–
Diabetes mellites	3.75 (1.16–12.1)	0.03	6.20 (1.01–38.00)	0.04
Smoking	0.44 (0.11–1.76)	0.25	–	–
Alcohol Consumption	0.50 (0.14–1.71)	0.27	–	–
Methylation				
*APOE*	0.80 (0.25–2.55)	0.70	0.72 (0.14–3.56)	0.68
Methylation				
*FACTOR XIII*	1.37 (0.38–4.88)	0.62	1.76 (0.30–10.10)	0.53

*ACOM anterior communicating artery; *PCOM* posterior communicating artery; *MCA* middle cerebral artery; *ICA* internal carotid artery

## Discussion

Aneurysmal subarachnoid haemorrhage is a complex disease condition in which both genetic and environmental factors play a key role. Aneurysmal re-bleeding and delayed cerebral ischemia (DCI) are two major complications following aSAH that have increased mortality and poor prognosis [21]. Poor outcome is mainly characterised by the degree of initial bleed, size of aneurysm and age of patients at disease onset, high blood pressure, and hyperglycemia [5]. The first-degree relatives of patients with aSAH are 3 to5fold risker of having this disease than the general population [22]. However, not much information is available on the genes involved. Epigenetics modification can occur throughout the development and differentiation and in response to environmental stimuli [23]. DNA methylation is an epigenetic modification that is important for the regulation of gene expression.

Atherosclerosis is one of the pathogenetic mechanism for the formation of aneurysm [6]. Disturbed balance of lipid accumulation is one of the reasons for atherosclerosis [24]. Hypermethylation in the promoter region of various gene has been identified with the association of atherosclerosis progression [25]. *APOE* gene expression have been widely investigated in multiple disease condition. The *APOE* gene plays a key role in lipid metabolism by binding to cell surface lipoprotein receptors [26]. The *APOE* gene promoter methylation can alter the gene regulation and function. In our study we found that 64% of patient population and 60% of control population has methylation on *APOE* promoter region. Similarly, study done by Kordi-Tamandani et al. on the association of *APOE* promoter methylation with Schizophrenia in Asian population showed 80% of control population has methylated promoter region [27]. There is a significant difference in the *APOE* promoter methylation status with the present study (*χ2 = 8.87; p value = 0.003).* None of the above studies showed any significant association of *APOE* promoter methylation with these diseases. In-vitro study showed that *APOE* promoter polymorphism (− 219 G/T) modifies *APOE* expression in vitro and influences the binding to the estrogen receptor [28]. There are studies which highlights the association of *APOE* isoforms (ε2,ε3,ε4) in the exon 4 region with the risk of aSAH [29–31].

Factor XIII A (*F13A*) is a transglutaminase enzyme that circulates in the plasma and has two catalytic A subunit and two carrier B subunit [32]. Study done by Lu et al. suggests that demethylation of promoter in vivo increases the transglutaminase activity and hypermethylation of the transglutaminase promoter in vitro suppressed its activity [33]. Human transglutaminase gene expression can be regulated by methylation. In-vitro study showed that human transglutaminase promoter has three CpG rich domains of which two were fully methylated [34]. The difference between *F13A* subunit and transglutaminase gene is, *F13A* subunit gene has exon 1 encoding at 5’untranslated region and exon 2 has translational start site where as transglutaminase has translational start site at exon 1 [35].

In our study 74% of patients had methylated *F13A* promoter. The transcriptional repression of *F13A* gene leads to fibrinolytic dysfunction like increased bleeding [36]. In platelets and monocytes, *F13A* is present as cellular Factor XIIIA (c*FXIII*) [32]. Cellular *FXIII* has a key role in the progression of atherosclerosis by activating monocytes and helps adhesion of monocytes to endothelium [37]. Genetic variants at a specific locus can influence both regional and distant DNA methylation. Val34Leu polymorphism in *F13A* gene present on exon 2 of A subunit showed association with the risk of aSAH in many population [18, 20, 38]. Val34Leu polymorphism increases the activation rate and affects the fibrin structure [39]. Genetic variation in *F13A* gene encoding epigenetic factors can modify the risk of neurological disease onset and progression. The genetic risk and functional outcome after aSAH can be monitored with the SNP analysis and DNA methylation studies.

The present study had a greater number of cases with methylated *F13A* promoter region. Smoking level was seen higher in the control samples when compared to cases. Smoking is considered as the most powerful environmental modifier of DNA methylation. DNA methylation is significantly lower in smokers than non-smokers. There is a positive correlation between smoking level and F13A levels. The negative correlation between DNA methylation and F13A levels may explain the reason for lower methylation levels in control samples.

aSAH outcome were assessed using GOS at a similar time point (3 months) after aSAH in all the participants. Participants were recruited from all the eligible patients from the study hospital with recent aSAH. Recruitment of participants were done with a reduced risk of selection bias and a range of independent variables were included in the study with reasonable follow up rate. *APOE* and *F13A* promoter methylation was not associated with poor outcome in our study. But in our study, age, diabetes mellitus and ACOM aneurysm were independently associated with poor outcome in post aSAH cohort.

There are reasons to suggest the methylated genes as a good prognostic marker. Some of these reasons include the following: (a) DNA is a stable molecule which can be easily extracted from body fluids and tissues (b) detection of methylation percentage can be considered as an important marker to check gene expression change (c) Methylation tests might have clinical importance since they are non-invasive [40]. Epigenetic biomarkers can be easily extracted from body liquids such as blood, saliva, or urine and used to identify and diagnose neurological disorders at the initial phase of disease [41]. Methylation at specific loci in DNA from peripheral blood leukocytes/whole blood was first reported in lung cancer [42]. In epigenetic epidemiology, DNA from peripheral blood can be used for methylation analysis when target tissue for diseases such as aSAH was not readily available [43]. Study done by Ma et al., (2015) suggests that there was no significant variability in *APOE* promoter methylation levels between brain and blood tissue [44]. Large prospective studies are required to understand whether *F13A* DNA methylation patterns in WBC represent *F13A* expression levels on the pathogenic pathways that links to disease end points.

## Conclusion

In conclusion, epigenetics is at an early stage, but it is a promising area of analysis in aSAH treatment and recovery. Our study enacts a crucial step to clarify the role of *APOE* and *F13A* promoter methylation status with risk of aSAH in a South Indian population. In future, the information about the relationship between epigenetic variations and the risk of aSAH as well as its complications can help in the discovery of new treatment and therapies.

## Data Availability

Data used for this study cannot be made publicly available because additional studies are currently under way using the same data set.

## Abbreviations

ACOM: Anterior communicating artery
aSAH: Aneurysmal subarachnoid haemorrhage;
CI: Confidence interval
F13A: Factor XIII a subunit
ICA: Internal carotid artery
MCA: Middle cerebral artery
OR: Odds ratio
PCOM: Posterior communicating artery
WFNS: World Federation of Neurological Surgeons
